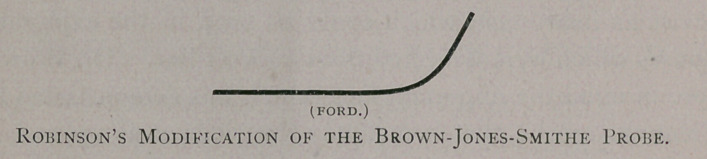# An Account of the Perineosinuexereeinator

**Published:** 1879-08

**Authors:** Jacques Robinson

**Affiliations:** Surgeon to the Hospital for Ruptured Vesicles, Member of the Anteversion Society, the Round-Ligament Club, etc.; Brownsville


### AN ACCOUNT OF THE PERINEOSINUEXEREEINATOR,

A NEW INSTRUMENT FOR THE EXPLORATION OF SINUSES—ESPECIALLY ADAPTED TO GYNECOLOGICAL PRACTICE

BY JACQUES ROBINSON, A. M., M. D., SURGEON, &C.

*To the Editor of the Louisville Medical News:*

I desire to call your attention to a new instrument which I have had the honor to devise, and through your columns to claim priority in its invention.

That I may be just to all parties, I may first state what were the evolutionary stages through which my instrument passed before it reached its present perfected shape.

Some years since—the exact date has escaped me—Dr. Smithe, the eminent gynecologist of Jonesville, gave to the professional world his since celebrated probe, a figure of which accompanies my text. This instrument has been known as the Smithe probe.

(GEMRIG.)

THE SMITHE PROBE.

It is three inches long, about the size of a knitting-needle, and is made of white metal. It has served an excellent purpose in the exploration of perineal sinuses; but it soon became evident that for sinuses which exceeded three inches in length the "Smithe probe" would not do. We are indebted to the genius of Dr. Jones, the renowned uterine pathologist of Smithville, for a solution of this difficulty. Dr. Jones modified the Smithe

(TIEMANN.)

THE JONES MODIFICATION OF THE SMITHE PROBE.

instrument so as to make it *four* inches long instead of three, thereby allowing sinuses of increased depth to be examined.

This was a great improvement, but the instrument was not yet perfect. Both the Jones and the Smithe instruments were confined in their operations to sinuses which were perfectly straight, and this fact led that obstetrical wonder, Dr. Brown, to devise an instrument which could be used in the exploration of sinuses which were deflected from a direct line. Dr. Brown also bore in mind the important fact, which was demonstrated by the Viennese school, that sometimes the sinus runs up and sometimes the sinus runs down. To meet this double difficulty he constructed a probe, which upon its right extremity ascends in a gentle curve, while upon its left extremity it descends in a similar manner. The accompanying diagram will illustrate these peculiarities perfectly, and will also show the capacity of the

BROWN'S MODIFICATION OF THE JONES-SMITHE PROBE.

instrument for deflection from the normo-rectal direction. In diagram 3 *a* represents one end of the probe and *b* the other. The other figures explain themselves.

It might have seemed, with these instruments before the profession, that hardly any thing more was to be desired for the convenience of the gynecologist; but my experience, which is by no means limited, has taught me that there are still objections to be urged to each of the instruments named, and I have endeavored—and I think that you will allow I have succeeded in my endeavor—to combine in one instrument the excellencies of all, with the imperfections of none.

The Smithe instrument was too short, the Jones modification was too straight, and the Brown modification was too curved, and, as will be seen at a glance, can be *only* used in deflected sinuses. I have therefore, after much experimentation, constructed an instrument, after the pattern in the accompanying

(FORD.)

ROBINSON'S MODIFICATION OF THE BROWN-JONES-SMITHE PROBE.

diagram, which it will be seen is curved at one end and straight at the other. If the sinus is straight, then the straight end is used; if the sinus is curved, the curved end is used. If it point upward, the curve is pointed in a similar direction; if it point downward, the curve is simply reversed (Q. E. D.) So, also, I have caused my instrument to be made of two sizes—one three inches long, the other four—that it might cover the same field with the Smithe instrument and the Jones modification.

I trust, Mr. Editor, that with this showing there will be no gainsaying that I have made a real advance in our art, and that hereafter no one will endeavor to claim my invention.

J. ROBINSON, M. D.,
*Surgeon to the Hospital for Ruptured Vesicles,*
*Member of the Anteversion Society,*
*the Round-Ligament Club, etc.*

BROWNSVILLE.

ILLUSTRATIVE CASES.

Since preparing for print an account of my new instrument—which I have named for convenience the Perineosinuexereeinator, the last paragraph of which being derived from the Greek word meaning "to explore"—a number of cases have occurred in my practice illustrating its usefulness in demonstrating both the presence and absence of sinuses. I select the following for publication:

Case I.—Mrs. A. B., aged forty years, female, brunette, bilious temperament, native of Kentucky, residence in Louisville, 397 West Thirty-Sixth Street, north side (up stairs); married 4th of July, 1866 (no cards); three children, named respectively Thomas, Richard, and Henry; weight, one hundred and twenty-three pounds (somewhat greater after eating.)

She states that her appetite is pretty good when she is hungry, generally sleeps at night, and is about during the day. Had suffered the week previous to her visit to me with perineal furuncle, for which ordinary remedies had been used, and it had discharged. Suspecting a sinus had resulted, I made exploration with the smaller of my instruments, and verified my diagnosis. Sinus measured .2 centimeter in depth. ℞ Argent. nit., to be used locally, and to take fluid ext. black haw. Cured.

Case II.—Mrs. MacF., Italian, aged fifty years, widow; occupation, attending clinics; parents dead; uncle living, also a number of cousins. Subject of retroflexion since birth of first child, thirty years previous. Has improved steadily under pessaries, which have been worn during the last ten years. Sinus suspected. None found. Diagnosis, chronic retroflexion. Treatment: hysterotomy (declined;) pessary continued; seabathing, and a trip to Europe.

Jacques Robinson, M. D.

## Figures and Tables

**Figure f1:**
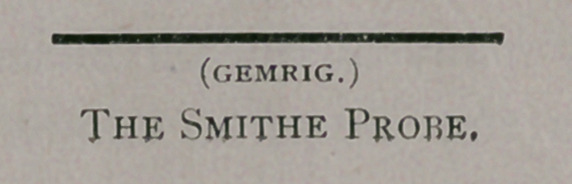


**Figure f2:**
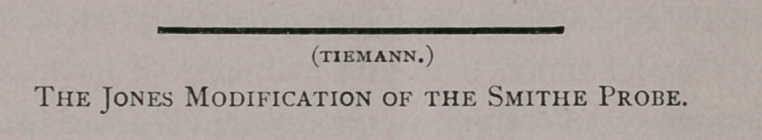


**Figure f3:**
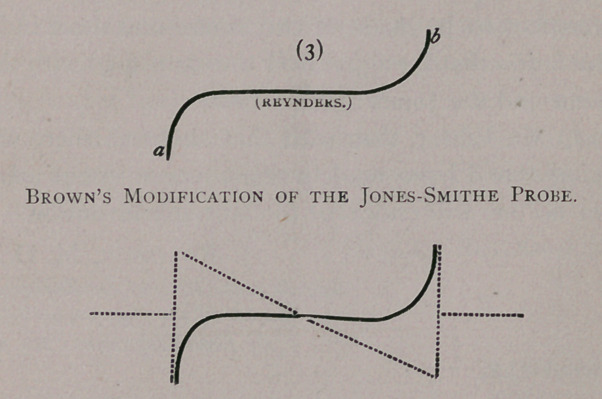


**Figure f4:**